# Endovascular therapy versus standard medical treatment for vertebrobasilar artery occlusion: a systematic review and meta-analysis

**DOI:** 10.3389/fneur.2026.1806643

**Published:** 2026-04-20

**Authors:** Qifan Zhang, Qing Zhao, He He, Kuang Yan, Jiarui Liu, Shunda Liu, Zhimin Shi, Aihua Liu

**Affiliations:** 1 People's Hospital of Ningxia Hui Autonomous Region, Third Clinical Medical College of Ningxia Medical University, Yinchuan, China; 2 Beijing Neurosurgical Institute, Beijing Tiantan Hospital, Capital Medical University, Beijing, China

**Keywords:** endovascular therapy, functional outcome, meta-analysis, standard medical treatment, vertebrobasilar artery occlusion

## Abstract

**Background:**

Acute vertebrobasilar artery occlusion (VBAO) is a devastating condition with high mortality and severe disability. Recent randomized controlled trials (RCTs) have significantly demonstrated that endovascular therapy is highly effective for VBAO, but its effectiveness for broader and real-world patient populations remains uncertain. This review aims to systematically evaluate the efficacy and safety of endovascular therapy for VBAO, integrating evidence from both RCTs and real-world data.

**Methods:**

We systematically searched PubMed, Embase, and the Cochrane Library from January 2000 to July 2025 for RCTs and observational cohort studies comparing endovascular therapy with standard medical treatment in patients with VBAO. Our primary outcome was 90-day favourable functional status, defined as a modified Rankin Scale score of 0 to 3. Secondary outcomes included functional independence (90-day modified Rankin Scale score of 0–2). Safety outcomes were symptomatic intracranial haemorrhage and 90-day mortality.

**Results:**

A total of 15 studies were included in the analysis, consisting of 4 RCTs (*n* = 988) and 11 observational cohort studies (*n* = 7,521). Compared with SMT, EVT was associated with a significantly higher likelihood of a favorable functional outcome (mRS score 0–3: OR = 1.92; 95%CI 1.51–2.43) and functional independence (mRS score 0–2: OR = 1.76; 95%CI 1.39–2.23), and a significantly lower risk of 90-day all-cause mortality (OR = 0.58; 95%CI 0.49–0.68). However, endovascular therapy increased the risk of symptomatic intracranial hemorrhage (OR = 2.57, 95%CI 1.31–5.06). Subgroup analyses showed that EVT was beneficial in patients with NIHSS ≥10, PC-ASPECTS <8, and onset-to-admission time >6 h (all *p* < 0.05). However, the treatment benefit was not significant in several subgroups, including elderly patients (≥75 years), those with mild deficits (NIHSS<10), distal basilar occlusion, and patients who received intravenous thrombolysis.

**Conclusion:**

This integrated analysis confirms endovascular therapy as a cornerstone treatment for acute vertebrobasilar artery occlusion, demonstrating significant improvements in functional outcomes and mortality despite increased symptomatic intracranial hemorrhage risk.

**Systematic review registration:**

https://inplasy.com/projects/, identifier INPLASY202590088.

## Introduction

Vertebrobasilar artery occlusion (VBAO) accounts for approximately 20% of all ischemic strokes, with high mortality and severe disability (1). Endovascular therapy (EVT) is now standard for anterior circulation large vessel occlusion (2, 3), but evidence for EVT in VBAO has accumulated more slowly. Case reports of intra-arterial thrombolysis with streptokinase date back to 1983 (4), yet a 2005 randomized controlled trial of urokinase for acute posterior circulation stroke enrolled only 16 patients (5). This trial was prematurely terminated due to low enrollment rates, resulting in an evidence gap that persisted for decades. Nevertheless, the findings suggested potential therapeutic value: good functional outcomes were observed in 4 of 8 patients in the treatment group, compared to 1 of 8 in the control group. Despite their limited scale, these early explorations highlighted both the substantial clinical need and the significant methodological challenges of rigorous research.

The subsequent publication between 2020 and 2022 of four pivotal randomized controlled trials (RCTs), including BEST (6), BASICS (7), ATTENTION (8), and BAOCHE (9), provided crucial evidence supporting the use of EVT in selected patients with VBAO. In the earlier BEST (6) and BASICS (7) trials, EVT showed a non-significant trend toward improved functional outcomes (mRS score 0–3) compared to medical management alone. Based on these foundations, the ATTENTION (8) and BAOCHE (9) trials more clearly demonstrated the superiority of EVT in patients with basilar artery occlusion. This was achieved through key methodological refinements, including efficient patient enrollment, low crossover, extended time windows, and the application of advanced imaging selection (10).

RCTs are considered the gold standard for evaluating the efficacy of interventions (11). However, their feasibility is often limited by factors such as ethical considerations, strict inclusion criteria (e.g., restricted populations, specific disease severity, or narrow treatment time windows), and highly controlled clinical settings (12). These constraints may fail to represent the broader patient populations in real-world practice. Therefore, to enhance the reliability and applicability of clinical evidence, it’s crucial to conduct RCTs with research based on real-world evidence (RWE). Our meta-analysis systematically integrates RCT efficacy evidence with cohort effectiveness findings, strengthening the evidence base for clinical decision-making, reconciling potential discrepancies between experimental and real-world outcomes, and ultimately providing a more generalizable assessment of the intervention value.

## Methods

### Study design and registration

This study adheres to the Preferred Reporting Items for Systematic Reviews and Meta-Analyses (PRISMA) guidelines (13). The study protocol was registered on the International Platform of Registered Systematic Review and Meta-Analysis Protocols (Registration No. INPLASY202590088), and last updated on December 2, 2025. As this study synthesized published data, patient consent was not applicable.

### Data sources and search strategy

A systematic search was conducted in PubMed, Embase, and the Cochrane Library from January 1, 2000 to July 1, 2025. The search strategy combined Medical Subject Headings (MeSH) terms and free-text keywords for vertebrobasilar artery occlusion (e.g., “Vertebrobasilar artery occlusion,” “Basilar artery occlusion,” “Posterior circulation stroke”) and endovascular therapy (e.g., “Thrombectomy,” “Endovascular treatment”). The complete search strategy for each database is provided in Supplementary material.

### Eligibility criteria

Studies were selected based on the PICO framework: (1) Population: adults (≥16 years) with acute vertebrobasilar artery occlusion (VBAO); (2) Intervention: Endovascular therapy (EVT), with or without intravenous thrombolysis (IVT); (3) Comparison: Standard medical treatment (SMT). Patients in each treatment group received the standard medical care, including IVT agents, antiplatelet drugs, anticoagulation, or combinations of these treatments according to the national and institutional guidelines (6). (4) Outcomes: The primary outcome was a favorable functional outcome (mRS score 0–3 at 90 days). Secondary outcomes included functional independence (mRS 0–2 at 90 days), sICH, and all-cause mortality at 90 days.

Primary exclusion criteria were as follows: (1) Single-arm studies; (2) Studies with any treatment group containing less than 10 patients; (3) Overlapping datasets and studies with missing outcomes data; (4) Non-English-language studies.

### Data extraction

In this study, two independent reviewers screened studies, extracted data, and resolved discrepancies through consensus. A standardized data extraction form was used to systematically collect information on study design, patient demographics, comorbidities, and confounder adjustments. The detailed distribution of comorbidities across individual studies is shown in Supplementary Table 1, while the specific confounders adjusted for in each study are outlined in Supplementary Table 2.

### Risk of bias assessment

The methodological quality of included studies was independently assessed by two reviewers. RCTs were evaluated using the Cochrane Risk of Bias (RoB) 2.0 tool, and observational studies underwent appraisal with the Newcastle-Ottawa Scale (NOS). Disagreements between reviewers were resolved through consensus discussion.

### Statistical analysis

To minimize confounding bias, the primary meta-analysis prioritized adjusted effect estimates, with covariates adjusted in each included study (including age, baseline National Institutes of Health Stroke Scale score, and comorbidities). Adjusted odds ratios (ORs) were directly utilized when available. For studies reporting adjusted risk ratios (RRs), RRs were converted to ORs using the following formula: OR ≈ RR × [(1 − p_₀_)/(1 − p_1_)], where p_₀_ and p_₁_ represent the observed event rates in the control and treatment groups, respectively (14). Studies lacking adjusted estimates (primarily due to low event rates prohibiting multivariate adjustment) were retained to mitigate potential selection bias; unadjusted ORs were calculated from raw 2 × 2 contingency tables for these studies. When studies reported multiple adjusted effect estimates, we consistently selected the most rigorously adjusted estimate according to a predefined hierarchy of adjustment methods. All source data for these meta-analytic outcomes are detailed in Supplementary Table 3.

The distribution of mRS outcomes was visualized with a sequential plot, synthesizing data from studies reporting original mRS ordinal data. mRS data were independently extracted from publications by 2 reviewers.

For the meta-analysis of treatment effect, the inverse-variance weighting method was applied to pool the odds ratios (ORs) across studies. Heterogeneity was assessed using the I^2^ statistic. A random-effects model was applied for all outcomes to account for between-study heterogeneity.

Pre-specified subgroup analyses were conducted for the primary outcome based on age, sex, baseline stroke severity (NIHSS), baseline Posterior Circulation Acute Stroke Prognosis Early Computed Tomography Score (pc-ASPECTS), intravenous thrombolysis (IVT) administration, occlusion site, and onset-to-admission (OTA) time. To maintain consistency with the original data reported across most studies in these subgroups, risk ratios (RRs) served as the effect measure for subgroup synthesis; detailed RR results are available in Supplementary Table 4.

Sensitivity analyses for the primary outcome were conducted using four approaches: (1) leave-one-out; (2) adjusted-OR only; (3) raw event data; (4) fixed-effect model. Publication bias was assessed visually using funnel plots and statistically using Egger’s and Begg’s tests. All analyses were performed using Stata 19.0, with a two-sided *p*-value <0.05 considered statistically significant (Table 1).

**Table 1:** Baseline characteristics of included studies.

Author (year)	Study region	Study duration	Study type	center	Symptom onset to inclusion (hours)	Confounding adjustment	Number (EVT|SMT)	Male sex no. (%)	Median age (EVT|SMT)	Median NIHSS (EVT|SMT)	Median pc-ASPECTS (EVT|SMT)	IVT(EVT) no. (%)	sICH no.(EVT|SMT)
Tao et al., 2022 (ATTENTION) (8)	China	2021–2022	RCT	36	0–12	Multivariable logistic regressions	228|114	231 (67.9)	66.0|67.3†	24 (15–35)|24 (14–35)	9 (8–10)|10(8–10)	69 (30.0)	12|0
Jovin et al., 2022 (BAOCHE) (9)	China	2016–2022	RCT	22	6–24	Logistic regression	110|108	159 (50.7)	65|64	20 (15–29)|19 (12–30)	8 (7–10)|8 (7–10)	15 (14.0)	9|2
Liu et al., 2020 (BEST) (6)	China	2015–2017	RCT	28	0–8	Multivariable regression	66|65	100(76.3)	62|68	32(18–38)|26 (13–37)	8 (7–9)|8 (7–9)	18 (27.0)	5|0
Langezaal et al., 2021 (BASICS) (7)	International	2011–2019	RCT	23	0–6	Multivariable regression	154|146	196(65.3)	66.8|67.2†	21.9|22.1†	10 (10–10)|10(10–10)	121 (79.0)	7|1
Zi et al., 2020 (BASILAR) (15)	China	2014–2019	PCS	47	0–24	PSM multivariable regression	647|182	617(73.8)	64|67	27 (17–33)|26.5 (16–33)	8 (7–9)|7 (6–8)	119(18.4)	45|1
Nicolin et al., 2024 (16)	International	2011–2021	PCS	NA	0–24	IPW	410|354	477 (62.4)	68.3 | 67.4†	5 (3–8)|6 (3–7)	NA	147 (36.9)	5|6
Liu et al., 2025 (17)	China	2019–2024	PCS	11	24–72	PSM IPTW	101|101	158 (78.2)	63|64	12(6–22)|10(7–17)	8 (7–9)|8 (8–10)	NA	4|0
Xu et al., 2024 (early time) (18)	China	2015–2022	RCS	65	0–6	PSM	1,425|1,048	1714 (69.3)	67|66	22 (13–29)|10 (5–24)	9 (7–10)|9 (8–10)	314 (22.0)	NA
Xu et al., 2024 (late time) (18)	China	2015–2022	RCS	65	6–24	PSM Multivariable logistic regressions	997|654	1,194 (72.3)	64|66	22 (13–28)|10 (5–24)	8 (7–10)|8 (7–10)	149(14.9)	NA
Chang et al., 2023 (19)	Korea	2008–2022	RCS	NA	0–24	IPTW PSM	314|252	339 (59.9)	73.2 |69.5†	20 (11–26)|16 (6–24)	5 (3–6)|5 (3–6)	119 (37.9)	8|7
Dargazanli et al., 2024 (20)	French	2012–2019	RCS	2	NA	IPTW	63|64	57 44.5%	63.4|69†	6 (4–6)|4(2–6)	8 (7–9)|8 (7–9)	15(38.5)	9|2
Yoshimoto et al., 2020 (mild) (21)	Japan	2014–2016	RCS	46	0–24	NA	18|21	32 (82.1)	77|69	7(5–9)|4 (3–5)	8 (7–9)|9 (7–9)	7 (38.9)	2|0
Yoshimoto et al., 2020 (severe) (21)	Japan	2014–2016	RCS	46	0–24	NA	111|21	83 (62.9)	75|80	28(21–33)|25(19–30)	7(6–8)|6 (4–10)	49 (44.1)	1|0
Raty et al., 2025 (24)	International	2010–2024	RCS	6	NA	IPWRA	372|151	339 (64.9)	69|71	15 (7–28)|11 (5–25)	10 (8–10)|10 (9–10)	208 (55.9)	19|9
Seners et al., 2021 (25)	French	2006–2018	RCS	45	NA	PSM	28|29	36 (63.2)	67|71	4(2–5)|4(2–5)	9 (8–10)|9 (8–10)	28(100)	NA
Dias et al., 2017 (21)	Brazil	2001–2011	RCS	1	NA	NA	19|44	41 (65.1)	65|64	29 (15–33)|39 (29~38)	8 (7–8)|7(5–9)	NA	1|1
Broussalis et al., 2013 (22)	Austria	2005–2012	PCS	NA	NA	NA	77|22	51 (51.5)	68|NA	22(4–28)|NA	NA	30 (38.9)	18|2

Continuous variables are described as median (interquartile range); categorical variables as numbers (percentages). † Denotes mean value. NA, not applicable; RCT, Randomized Controlled Trial; PCS, Prospective Cohort Study; RCS, Retrospective Cohort Study; IVT, intravenous thrombolysis; NIHSS, National Institutes of Health Stroke Scale; pc-ASPECTS, posterior circulation Acute Stroke Prognosis Early CT Score; EVT, endovascular therapy; SMT, standard medical treatment; IPWRA, inverse probability-weighted regression adjustment; PSM, Propensity Score Matching; IPTW, Inverse Probability of Treatment Weighting; IPW, Inverse Probability Weighting.

## Results

### Literature search

**Figure 1:**
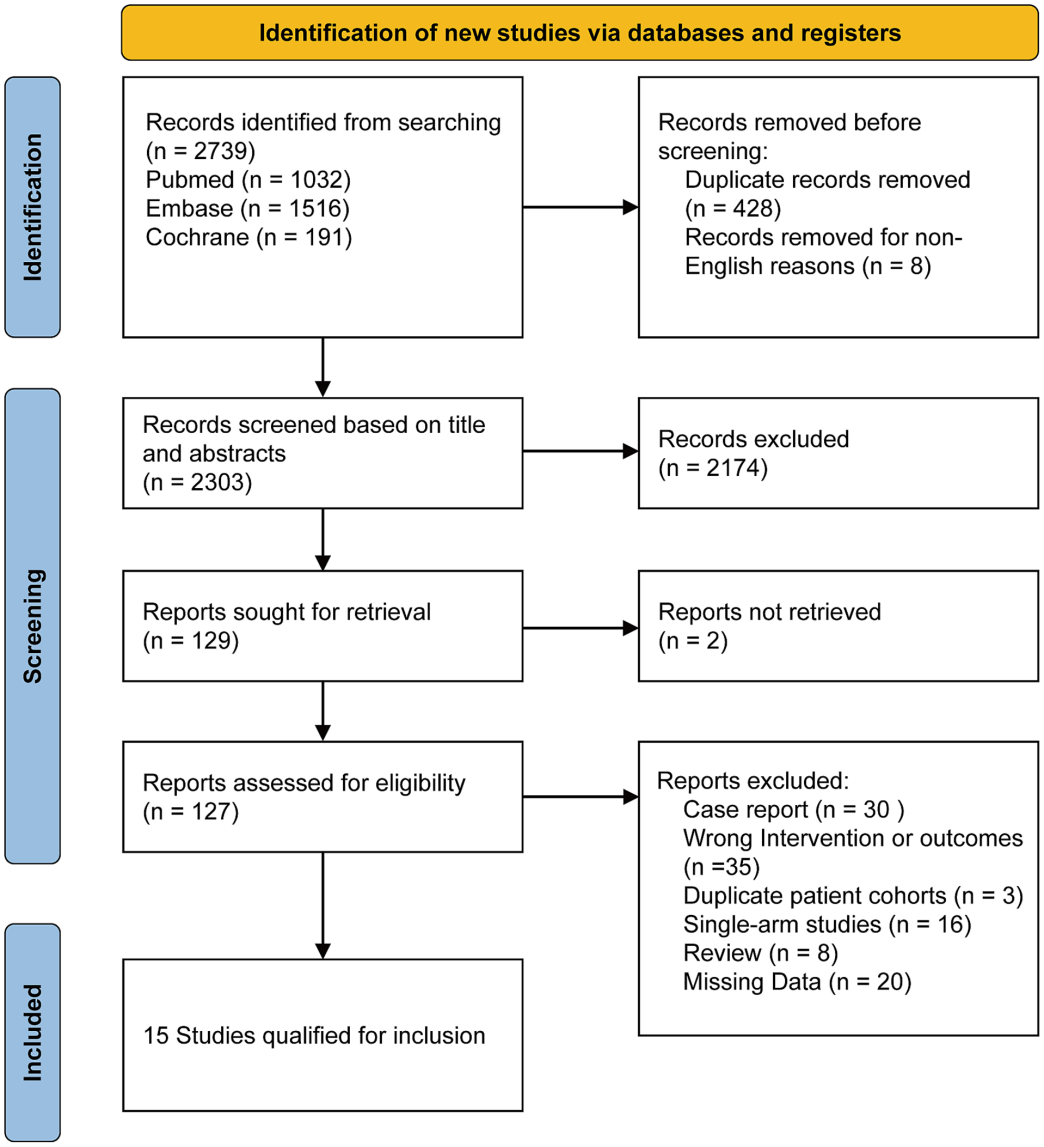
Flowchart of literature search and study selection.

A total of 2,739 relevant studies were initially screened according to the search strategy. After further removing 428 duplicates and 8 non-English records, 2,303 studies underwent title and abstract screening. Of these, 2,174 were excluded and two more were excluded for inability to retrieve the full text, leaving 127 studies for full-text assessment. Then, 112 studies were excluded for specific reasons such as duplicate data, irrelevant outcomes and so on. Consequently, 15 studies were included in the final meta-analysis. The study selection process is detailed in the PRISMA flow diagram (Figure 1).

### Study characteristics

The meta-analysis included 15 studies, containing 4 RCTs (*n* = 988) (6–9) and 11 observational studies (*n* = 7,521) (15–25). Notably, 2 of the included studies, Xu et al. (18) and Yoshimoto et al. (19) reported stratified analyses based on symptom severity or time window. Since these analyses represent subgroups within individual studies rather than independent studies, each was counted as a single study in the total (Table 1).

Among the 988 patients included from 4 RCTs (6–9), key baseline characteristics were as follows: the median age ranged from 62 to 68 years, 69% of the population was male, the median NIHSS scores ranged from 19 to 32, and median pc-ASPECTS values were between 8 and 10.

Eleven cohort studies enrolled a total of 7,521 patients across multiple countries (15–25). Compared with the RCTs, these cohorts exhibited greater clinical heterogeneity in their baseline profiles, including a wider median age range (63–80 years), a broader spectrum of initial stroke severity (median NIHSS score: 4–39), and a more varied distribution of pc-ASPECTS scores (5–10), despite a predominantly male population (79%). Consequently, most studies employed statistical adjustments for baseline imbalances. Furthermore, several studies applied advanced methodologies such as propensity score matching (PSM) and inverse probability weighted regression adjustment (IPWRA) to achieve baseline balance between groups (see Supplementary Table 2 for details). The exceptions were two early, small-sample studies Dias et al. (21) and Broussalis et al. (22), which neither reported methods for confounding adjustment nor provided adjusted effect estimates.

### Primary outcome: favorable functional outcome (90-day mRS score 0–3)

1. Evidence by study design

**Figure 2:**
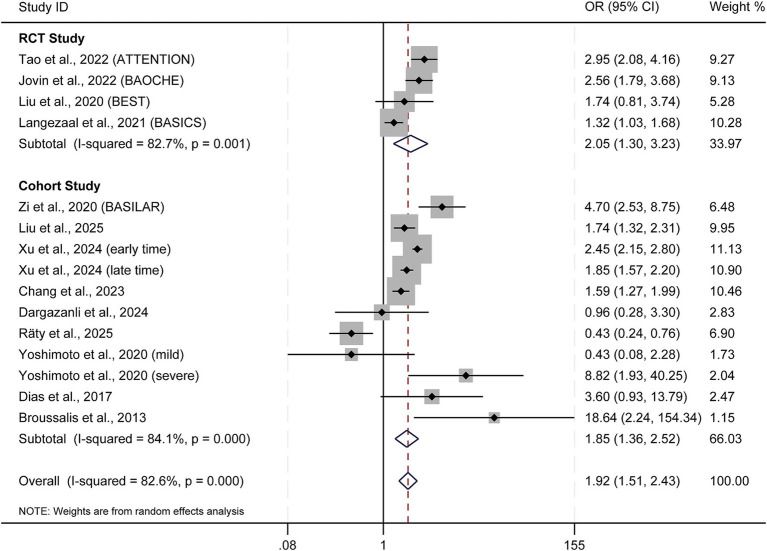
Forest plot of 90-day favorable functional outcome (mRS score 0–3). OR, odds ratio; CI, confidence interval.

Pooled analysis across four RCTs confirmed that EVT significantly improved favorable functional outcomes compared to SMT (OR = 2.05; 95% CI: 1.30–3.23; *p* = 0.002; I^2^ = 82.7%) (Figure 2). This compelling benefit was consistent in the pooled analysis of eleven observational cohort studies (OR = 1.85; 95% CI: 1.36–2.52; *p* < 0.001; I^2^ = 84.1%). When all 15 studies were combined, the overall pooled estimate confirmed the robust efficacy of EVT (OR = 1.92; 95% CI: 1.51–2.43; *p* < 0.001; I^2^ = 82.6%).

1. Sensitivity analyses

The pooled effect estimate maintained statistical significance and consistent direction when subjected to multiple sensitivity analyses, leave-one-out analysis, analysis using a fixed-effect model, and analysis restricted to adjusted odds ratios. Notably, the meta-analysis based solely on unadjusted data, predominantly from observational cohorts—produced a concordant but less precise benefit (OR = 1.51; 95% CI: 1.02–2.24; *p* = 0.040). Detailed results of these analyses are presented in Supplementary Figures 2–5.

### Secondary outcome: functional independence (90-day mRS score 0–2)

**Figure 3:**
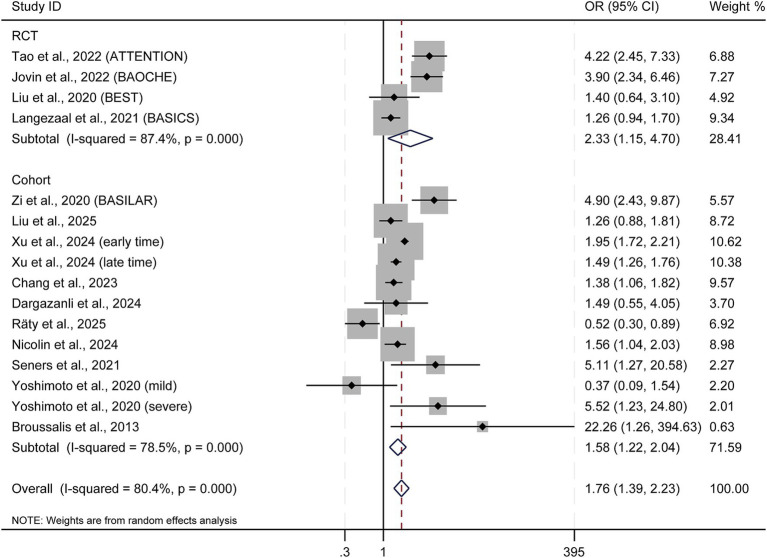
Forest plot of 90-day functional independence outcome (mRS score 0–2). OR, odds ratio; CI, confidence interval.

In the four included RCTs, EVT demonstrated significantly higher odds of achieving 90-day functional independence versus medical management (OR = 2.33; 95% CI: 1.15–4.70; *p* = 0.019; I^2^ = 87.4%), as shown in Figure 3. This was supported by observational studies (OR = 1.58; 95% CI: 1.22–2.04; *p* = 0.001; I^2^ = 78.5%). The overall effect remained significant in the pooled analysis (OR = 1.76; 95% CI: 1.39–2.23; *p* < 0.001; I^2^ = 80.4%).

### Safety outcomes: symptomatic intracerebral hemorrhage (sICH) and 90-day mortality

1. Symptomatic intracerebral hemorrhage (sICH)

**Figure 4:**
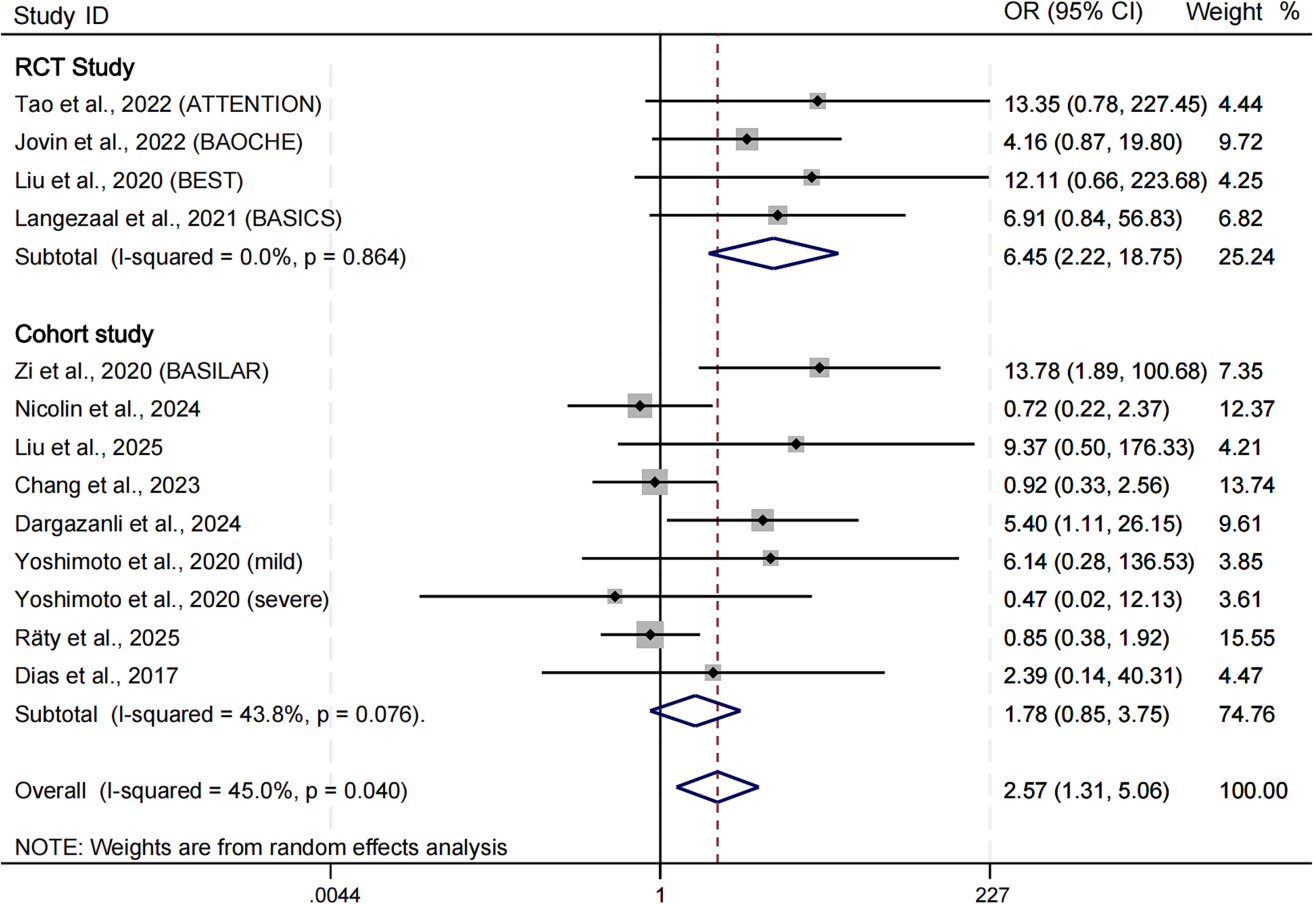
Forest plot of symptomatic intracerebral hemorrhage (sICH). OR, odds ratio; CI, confidence interval.

Across the four RCTs, EVT demonstrated a significantly elevated risk of sICH compared to SMT(OR = 6.45; 95% CI: 2.22–18.75; *p* = 0.001; I^2^ = 0.0%) (Figure 4). A similar but non-significant elevation was observed in the cohort studies (OR = 1.78; 95% CI: 0.85–3.75; *p* = 0.129; I^2^ = 43.8%). When all studies were pooled, the increased risk remained significant (OR = 2.56; 95% CI: 1.31–5.06; *p* = 0.006; I^2^ = 45.0%).

1. 90-day mortality

**Figure 5:**
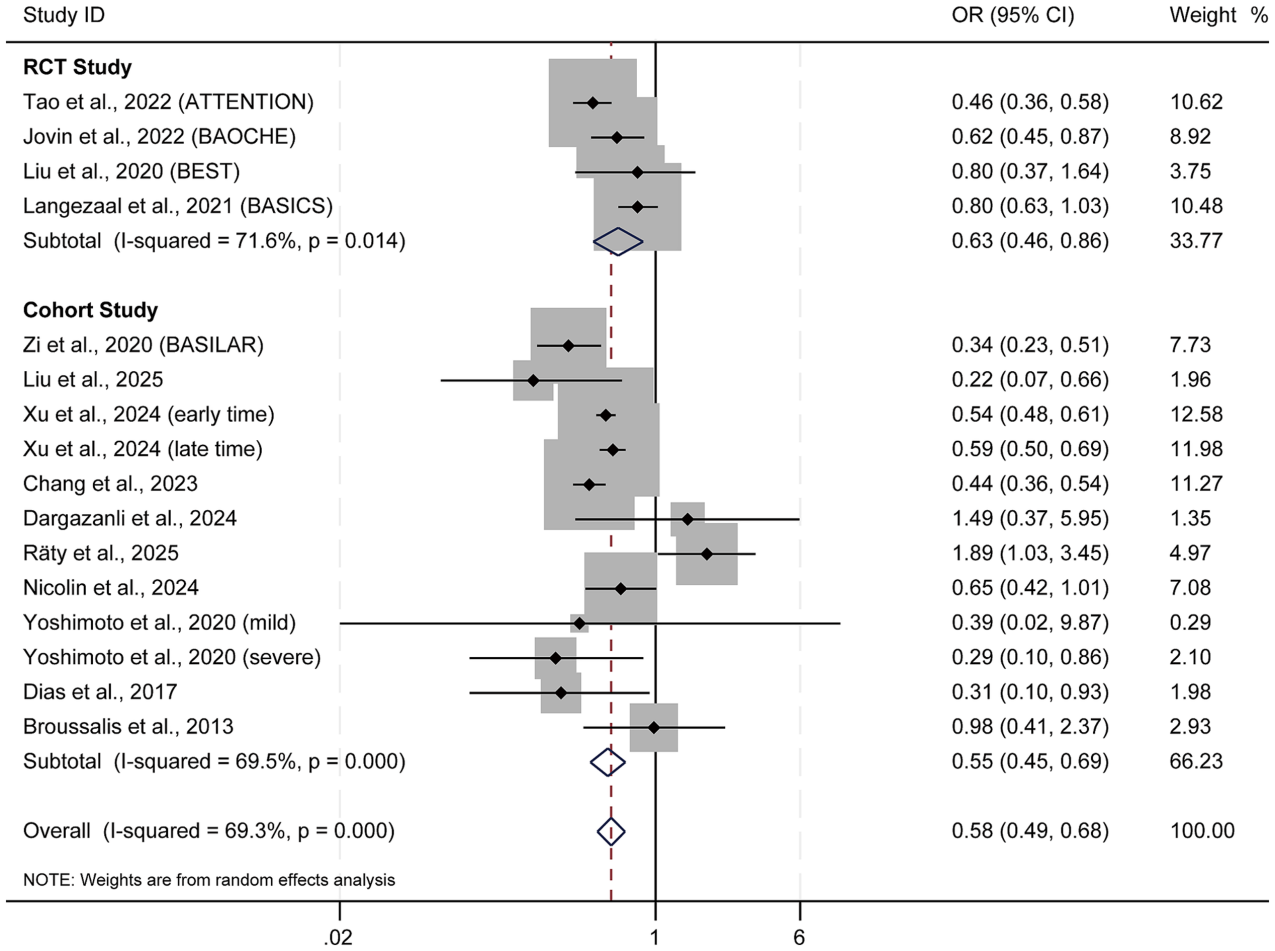
Forest plot of 90-day mortality. OR, odds ratio; CI, confidence interval.

EVT dramatically reduced 90-day mortality compared to SMT in RCTs (OR = 0.63; 95% CI: 0.46–0.86; *p* = 0.003; I^2^ = 71.6%) (Figure 5), with a comparable benefit observed in cohort studies (OR = 0.55; 95% CI: 0.45–0.69; *p* < 0.001; I^2^ = 69.5%). Overall, the pooled analysis revealed a substantial reduction in mortality with EVT (OR = 0.58; 95% CI: 0.49–0.68; *p* < 0.001; I^2^ = 69.3%).

### Subgroup analyses

**Figure 6:**
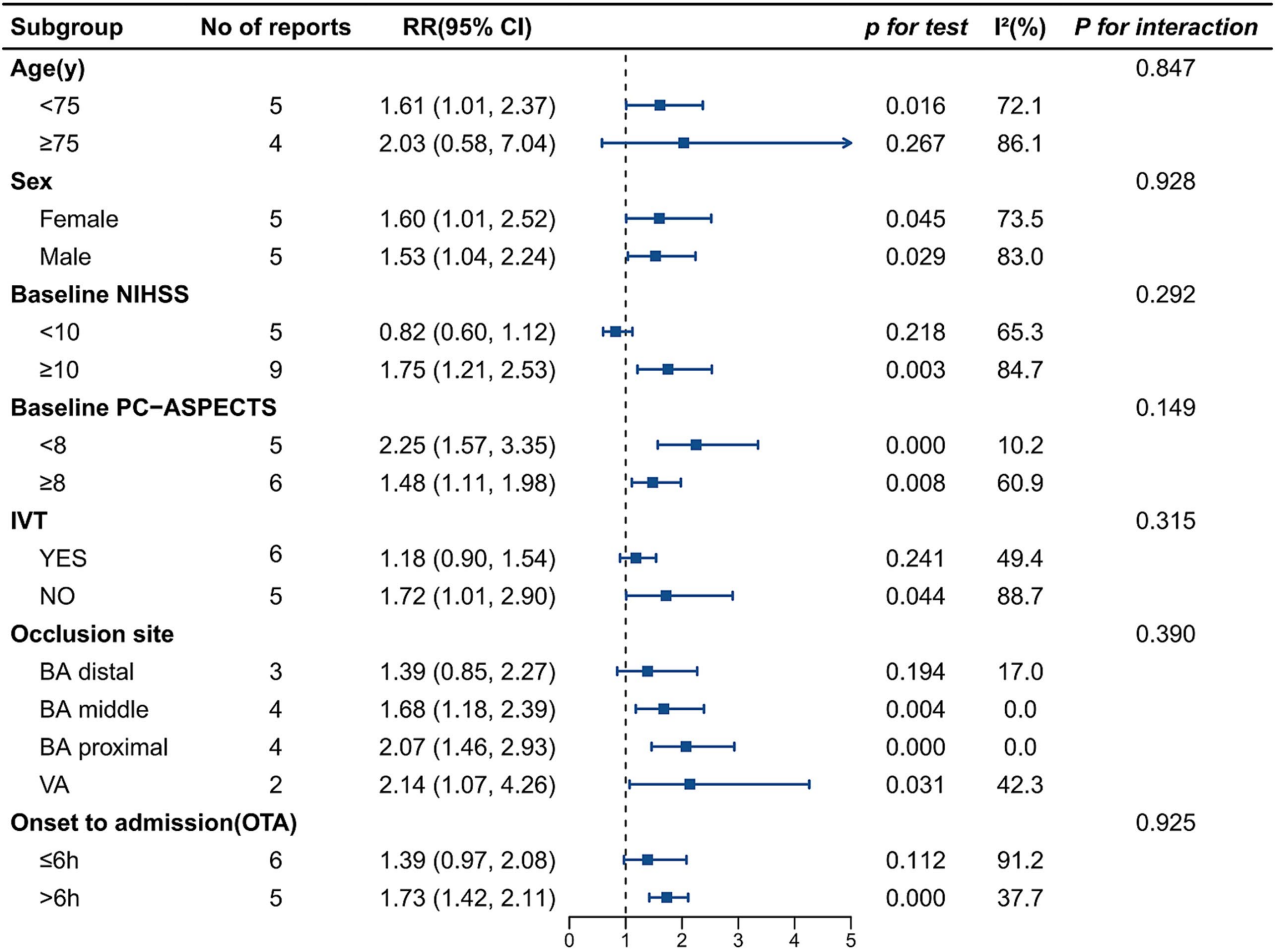
Subgroup analysis for 90-day favorable functional outcome (mRS score 0–3). RR, risk ratio; CI, confidence interval; *I*^2^, heterogeneity statistic; *p* for interaction, probability value for interaction test; EVT, endovascular therapy; SMT, standard medical treatment; VBAO, vertebrobasilar artery occlusion; mRS, modified Rankin Scale; NIHSS, National Institutes of Health Stroke Scale; PC-ASPECTS, posterior circulation Alberta Stroke Program Early CT Score; IVT, intravenous thrombolysis; OTA, onset to admission.

The results of the subgroup analyses for the primary outcome (90-day mRS score 0–3) are presented in Figure 6. We assessed heterogeneity of treatment effect across the pre-specified variables, including age, sex, baseline stroke severity (NIHSS), baseline pc-ASPECTS, IVT use, occlusion site, and onset-to-admission (OTA) time. The direction of effect favored endovascular therapy across all strata except for baseline NIHSS below 10, although the RR for treatment was not significant for patients age ≥75 years, those who received IVT, distal basilar artery occlusion, and onset-to-admission time within 6 h. Remarkably, in several key subgroups, EVT delivered impressive and statistically significant benefits. This was particularly evident among patients with moderate-to-severe stroke (NIHSS≥10: RR = 1.75; 95% CI 1.21–2.53; *p =* 0.003), those exhibiting more extensive baseline infarction (pc-ASPECTS <8: RR = 2.25; 95% CI 1.57–3.35; *p* < 0.001), and individuals suffering from proximal basilar artery (BA) occlusion (RR = 2.07; 95% CI 1.46–2.93; *p* < 0.001).

### Risk of bias and publication bias

The overall methodological quality of the included studies was high. The ATTENTION and BAOCHE trials had a low risk of bias across all domains. The BEST trial was assessed as having a high risk of bias, primarily attributed to deviations from the intended intervention, whereas the BASICS trial raised some concerns in the same domain. All observational studies achieved high Newcastle-Ottawa Scale scores (range: 7–9; majority ≥8), indicating good overall quality (Supplementary Tables 5, 6).

Regarding publication bias for the primary outcome, no substantial evidence was detected. Visual inspection of the funnel plot revealed symmetry (Supplementary Figure 6). which was further supported by formal statistical tests (Egger’s test: *p* = 0.869; Begg’s test: *p* = 0.805).

### Supplementary analyses

Supplementary analyses further supported the main findings. The ordinal analysis of mRS scores showed a consistent shift toward better outcomes with EVT across the entire outcome distribution (Supplementary Figure 1). Across all studies, the proportion of patients with favorable outcome was 46.1% in the endovascular therapy group, compared with 36.1% in the standard medical treatment group. In subgroup analyses, favorable outcome rates were 45.2% vs. 29.6% in randomized controlled trials, and 54.5% vs. 37.3% in cohort studies for the endovascular and standard treatment groups, respectively.

## Discussion

This meta-analysis, incorporating 4 RCTs and 11 real-world cohort studies, provides comprehensive evidence supporting the efficacy and safety of EVT for VBAO. The principal finding is that EVT significantly improves functional outcomes and reduces mortality at 90 days compared to medical management alone, despite an associated increase in the risk of sICH, confirming a robust net clinical benefit.

In RCTs, EVT improved the odds of favorable functional outcome (mRS 0–3) by 2.05-fold and functional independence (mRS 0–2) by 2.33-fold compared with medical therapy alone. The success of ATTENTION (8) and BAOCHE (9) trials, in contrast to earlier neutral results of BEST (6) and BASICS (7), can be attributed to three key optimizations: high randomization with low crossover rates, refined patient selection (baseline NIHSS≥10), and extended treatment windows (up to 24 h) with adjunct interventions like angioplasty and stenting (26, 27). Real-world evidence confirmed these benefits, showing 1.58-fold (mRS 0–2) and 1.85-fold increased likelihood of functional independence and favorable outcomes (mRS 0–3), respectively. Substantial heterogeneity among real-world studies (I^2^ = 84.1%) likely stems from variations in study design, inclusion criteria, stroke severity thresholds, and clinical EVT protocols (28).

In terms of safety outcomes, EVT significantly reduced mortality across both RCTs and cohort studies. Regarding symptomatic intracranial hemorrhage, while both study types indicate an elevated risk, yet their assessments differ: RCTs, employing prospective and standardized imaging adjudication criteria, show a statistically significant increase with high consistency across trials, whereas cohort studies showed a non-significant trend, likely due to symptom-driven imaging evaluation and broader definitions. This discrepancy does not diminish the clear net benefit of endovascular therapy.

Subgroup analyses revealed no significant interactions for any variable (all *p* for interaction >0.05). reinforcing the robust applicability of EVT. Several high-risk subgroups demonstrated particularly substantial benefits.

The Posterior Circulation Acute Stroke Prognosis Early Computed Tomography Score (pc-ASPECTS) is a core imaging indicator for clinical decisions on the suitability of endovascular therapy. Remarkably, patients with more extensive baseline infarction (pc-ASPECTS <8) demonstrated significantly greater therapeutic benefit, aligning with emerging anterior circulation evidence (29). Supporting this, Chang et al. (19) conducted a retrospective cohort study demonstrating a significant therapeutic benefit of endovascular therapy in patients with pc-ASPECTS ≤ 6. Notably, this therapeutic benefit was more pronounced in patients not receiving IVT. In contrast, trials such as BASICS (7) failed to show a statistically significant advantage of endovascular therapy over standard medical treatment, should not exclude patients from endovascular therapy eligibility. Future trials in this high-risk population should use advanced imaging for selection and endpoints beyond functional independence.

In the NIHSS subgroup analysis, EVT showed clear benefit in severe strokes (NIHSS≥10) but more modest effects in mild deficits (NIHSS<10). The NIHSS is inherently weighted toward anterior circulation deficits, which may underestimate posterior circulation severity. Whether EVT benefits patients with NIHSS <10 remains unanswered, limited by small sample sizes and favorable natural histories under medical therapy (20, 21). Notably, a study by Sun et al. (30) including 1,365 VBAO patients with NIHSS ≤10 found no overall EVT benefit (adjusted OR 1.17; *p* = 0.25). However, early EVT (door-to-puncture time ≤120 min) improved outcomes (OR 1.41; *p* = 0.02), whereas late EVT (>120 min) did not (OR 0.83; *p* = 0.39). This suggests that time-critical intervention may benefit even mild-to-moderate symptoms presentations.

Subgroup analyses suggested that endovascular therapy conferred a meaningful clinical benefit even when initiated beyond 6 h from symptom onset. In a study by Liu et al. (17) with median onset-to-admission of 48 h and rigorous imaging selection, EVT achieved higher favorable outcomes (57.7% vs. 45.1%) and lower mortality. Recent trials of late-window endovascular therapy for anterior circulation stroke have reported comparable outcomes, supporting a paradigm shift from rigid time windows to an imaging-guided selection strategy. Patients with favorable imaging profiles should not be excluded based on time alone (31). Conversely, caution is warranted for onset-to-admission time ≤6 h, where benefit did not reach statistical significance and heterogeneity was substantial (I^2^ = 91.2%).

Across all VBAO occlusion sites, our subgroup analysis demonstrated a consistent favorable trend for EVT. Specifically, EVT showed statistically significant benefits in proximal basilar artery occlusion, with non-significant trends in distal occlusion. Distal occlusions, often stemming from cardioembolic origins and presenting with more dramatic symptoms, exhibit a higher likelihood of spontaneous or medication-facilitated recanalization, which diminished the incremental advantage offered by EVT (32); additionally, this subgroup was under-powered due to limited sample sizes and substantial heterogeneity (I^2^ = 79%).

Patients aged ≥75 years displayed favorable but non-significant trends, potentially reflecting poor collaterals, reduced neuroplasticity, and comorbidity burden (e.g., atrial fibrillation, hypertension, diabetes, dementia) (33). Besides, EVT showed a reduced magnitude of benefit in patients who had received IVT (*p* = 0.241) versus those who had not (*p* = 0.044). This may reflect partial vascular recanalization from IVT diminishing endovascular treatment’s incremental benefit (34), or the influence of unmeasured subgroup confounding.

Compared with prior meta-analyses that primarily synthesized data from randomized trials (27), our study integrated RCT efficacy data with broad real-world effectiveness evidence. This comprehensive analysis bridges the traditional efficacy-effectiveness gap and strengthens the applicability to routine clinical practice.

### Limitations

Several limitations of this meta-analysis warrant consideration. First, considerable heterogeneity persisted, likely stemming from unmeasured confounders and inherent variations in real-world clinical practice across the observational cohorts. Second, the limited number of studies, particularly within specific subgroups, constrains the statistical power of our analyses, including tests for interaction and safety outcomes. Third, the potential for residual confounding exists, given differences in data availability and the statistical methods used for adjustment across studies. Fourth, inconsistent definitions of safety outcomes—especially symptomatic intracranial hemorrhage—across studies precluded a more precise estimation of risk. Finally, our analysis was confined to 90-day outcomes, leaving the long-term benefit of EVT uncertain.

## Conclusion

In conclusion, this integrated meta-analysis of randomized and real-world data establishes endovascular therapy as a standard, effective intervention for acute vertebrobasilar artery occlusion, significantly improving functional outcomes and reducing mortality despite an increased risk of symptomatic intracranial hemorrhage. However, the treatment benefit was less certain in several subgroups—including elderly patients (≥75 years), those with mild deficits (NIHSS<10), distal basilar occlusion, and patients receiving intravenous thrombolysis—where statistical significance was not reached, likely due to limited sample sizes or true effect modification. Future research should prioritize randomized controlled trials specifically designed for these underrepresented populations to validate the role of EVT and to establish optimal imaging and clinical selection criteria.

## Data Availability

The original contributions presented in the study are included in the article/Supplementary material, further inquiries can be directed to the corresponding author.
