# Supplementary material for: Endovascular therapy versus standard medical treatment for vertebrobasilar artery occlusion: a systematic review and meta-analysis
Source: Front Neurol. 2026 Apr 20;17:1806643. doi: 10.3389/fneur.2026.1806643 (PMC13137881; doi:10.3389/fneur.2026.1806643)
Supplement: Supplementary file 1 [file Table_1.docx]

Supplementary Information for

Endovascular therapy versus standard medical treatment for vertebrobasilar artery occlusion: a systematic review and meta-analysis

**This PDF file includes:**

Supplementary Methods

Supplementary Figures:6

Supplementary Tables:6

****Supplementary Methods****

****Detailed Search Strategies****

**Search was conducted on [September 20, 2025].**

****PubMed****

Search Query:

#1 basilar artery occlusion[MeSH Terms]

#2 vertebral artery occlusion[MeSH Terms]

#3 **vertebrobasilar artery occlusion[MeSH Terms]**

#4 posterior circulation stroke[MeSH Terms]

#5 #1 OR #2 OR #3 OR #4

#6  percutaneous thrombectomy[MeSH Terms]

#7 intra-arterial therapy

#8 **endovascular treatment**

#9 **mechanical thrombectomy**

#10 **endovascular therapy**

#11 **endovascular surgery**

#12 #6 OR #7 OR #8 OR #9 OR #10 OR #11

#13 #5 AND #12

#14 #5 AND #12 Filters: from 2000/1/1 - 2025/7/1

**Results retrieved: 1,034**

****Embase****

**1.Intervention Concepts:**

**'percutaneous thrombectomy'/exp OR 'endovascular surgery'/exp OR 'endovascular therapy' OR 'endovascular treatment' OR 'intra-arterial therapy' OR 'mechanical thrombectomy'**

**2.Condition Concepts:**

**'basilar artery occlusion'/exp OR 'vertebral artery occlusion'/exp OR 'vertebrobasilar artery occlusion' OR 'posterior circulation stroke'/exp**

**3.Combination and Filters: #1 AND #2 AND [01-01-2000]/sd NOT [02-07-2025]/sd**

**Results retrieved: 1,516**

**Cochrane Library**

#1 MeSH descriptor: [Vertebrobasilar Insufficiency] explode all trees 100

#2 (Basilar Artery Occlusion):ti,ab,kw OR (vertebral artery occlusion):ti,ab,kw OR (vertebrobasilar artery occlusion):ti,ab,kw OR (posterior circulation stroke):ti,ab,kw (Word variations have been searched) 518

#3 MeSH descriptor: [Endovascular Procedures] explode all trees 13959

#4 MeSH descriptor: [Thrombectomy] explode all trees 861

#5 (endovascular surgery):ti,ab,kw OR (endovascular therapy):ti,ab,kw OR (endovascular treatment):ti,ab,kw OR (intra-arterial therapy):ti,ab,kw (Word variations have been searched) 4462

#6 #1 OR #2 580

#7 #3 OR #4 OR #5 17040

#8 #6 AND #7 with Cochrane Library publication date Between Jan 2000 and Jul 2025 191

**Results retrieved: 191**

****Supplementary Figures****

****Supplementary Figure 1. Distribution of functional outcomes (90-day mRS)****

**This analysis of the ordinal distribution of mRS scores was performed using the raw data from the following studies that reported the complete breakdown of mRS categories at 90 days: Tao et al., 2022 (ATTENTION); Jovin et al., 2022 (BAOCHE); Liu et al., 2020 (BEST); Langezaal et al., 2021 (BASICS); Zi et al., 2020 (BASILAR);Yoshimoto et al., 2020;Chang et al., 2023;Dargazanli et al., 2024;Yoshimoto et al., 2020;Nicolin et al., 2024.**

**
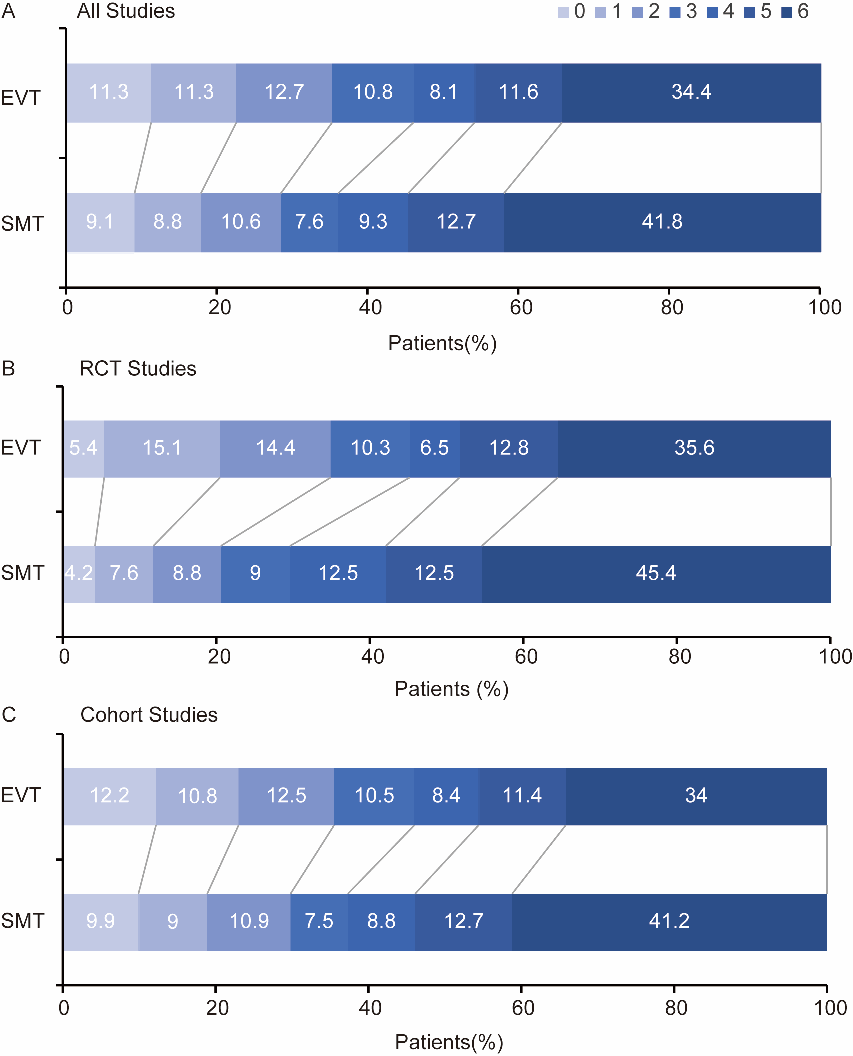
**

****Supplementary Figure 2. Leave-One-Out Sensitivity Analysis for the Primary Outcome (90-day mRS 0-3)****

****
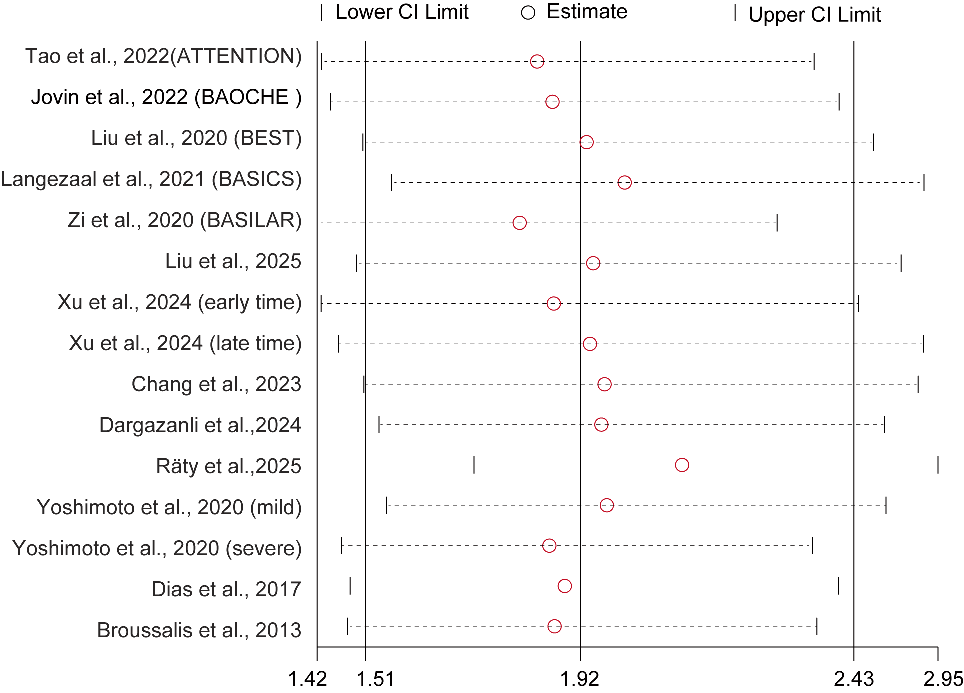
****

****Supplementary Figure 3. Adjusted OR Sensitivity Analysis for the Primary Outcome (90-day mRS 0-3)****

****
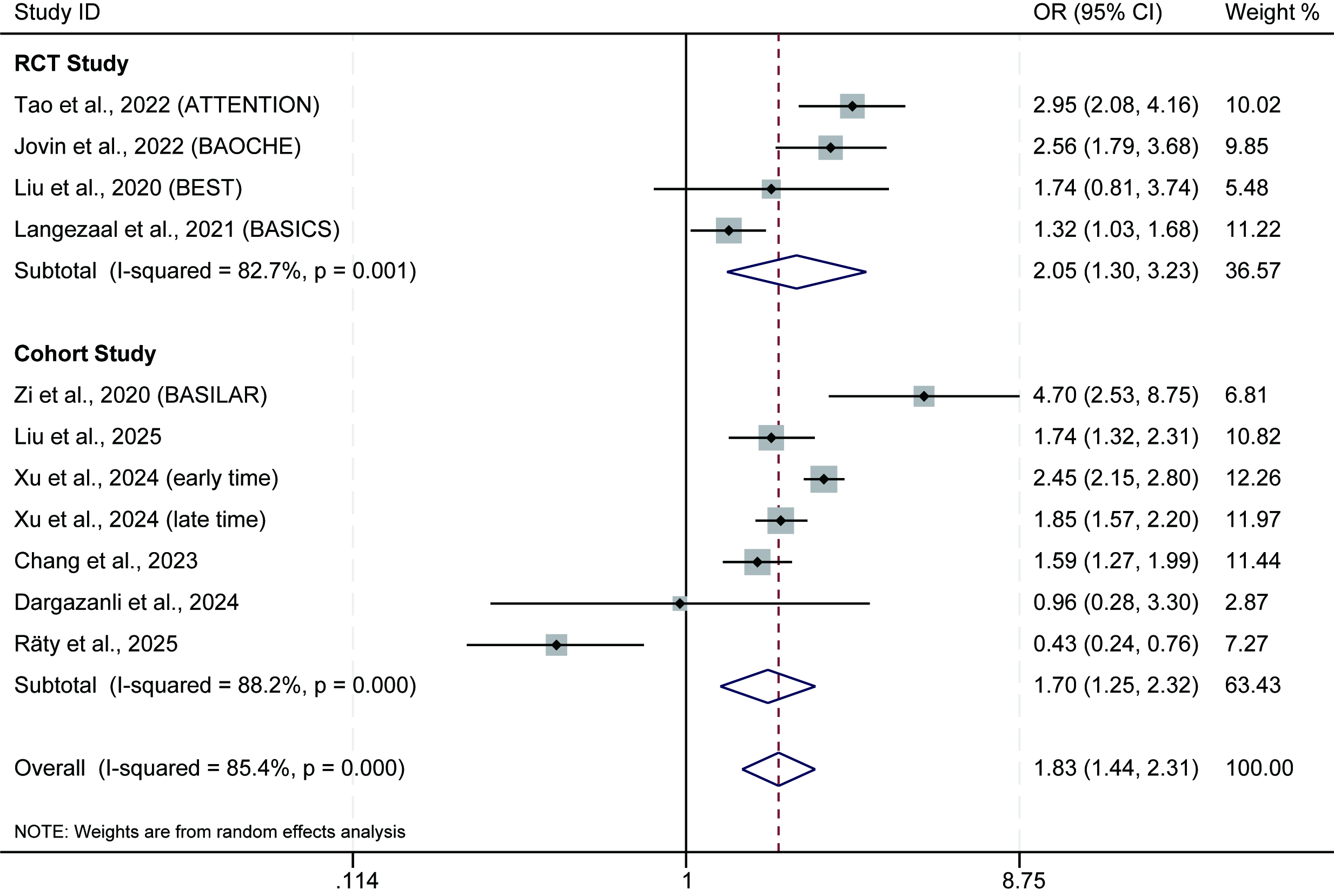
****

****Supplementary Figure 4. Fixed-Effect Model Sensitivity Analysis for the Primary Outcome (90-day mRS 0-3)****

****
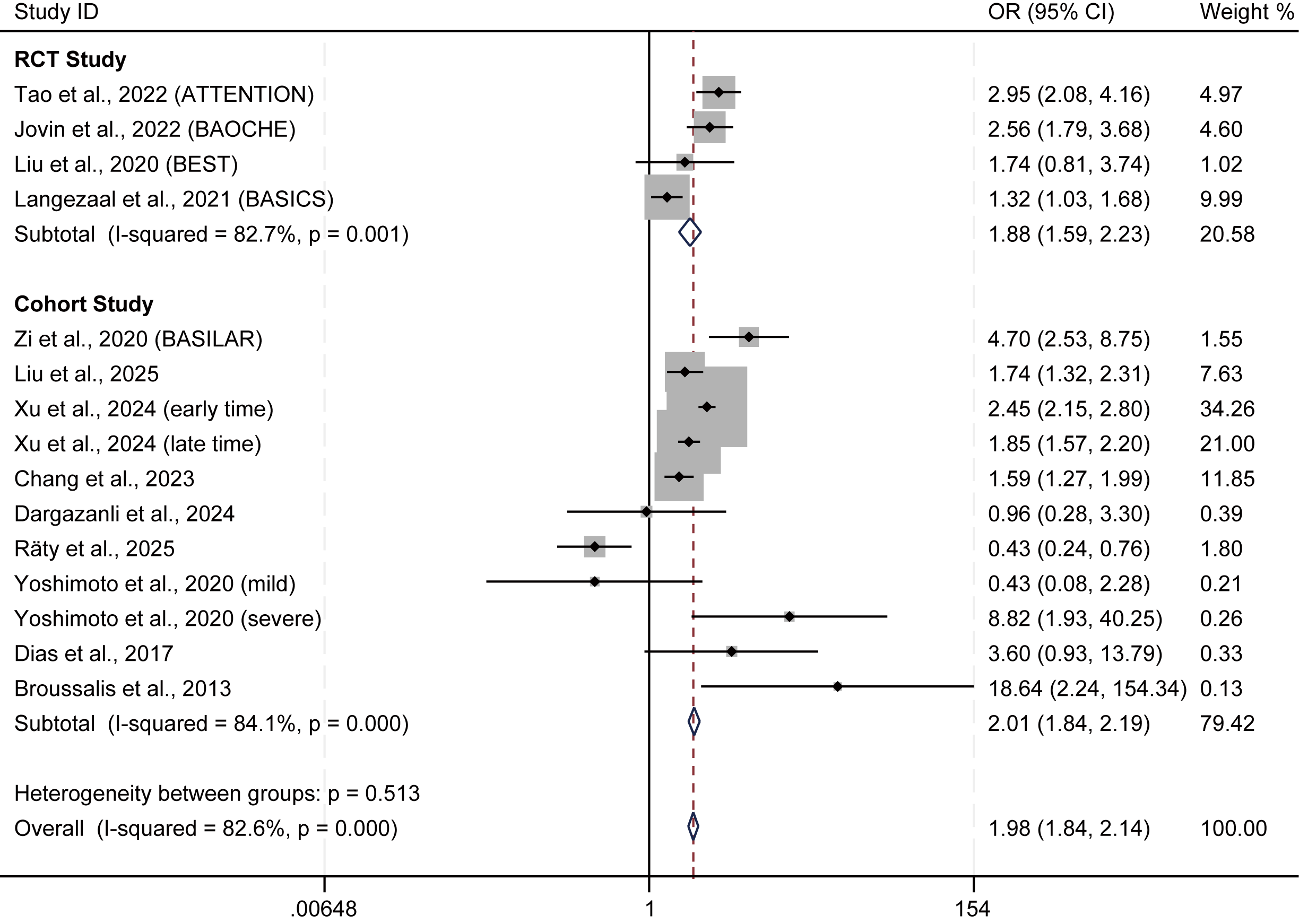
****

****Supplementary Figure 5. Unadjusted OR Sensitivity Analysis for the Primary Outcome (90-day mRS 0-3)****

****
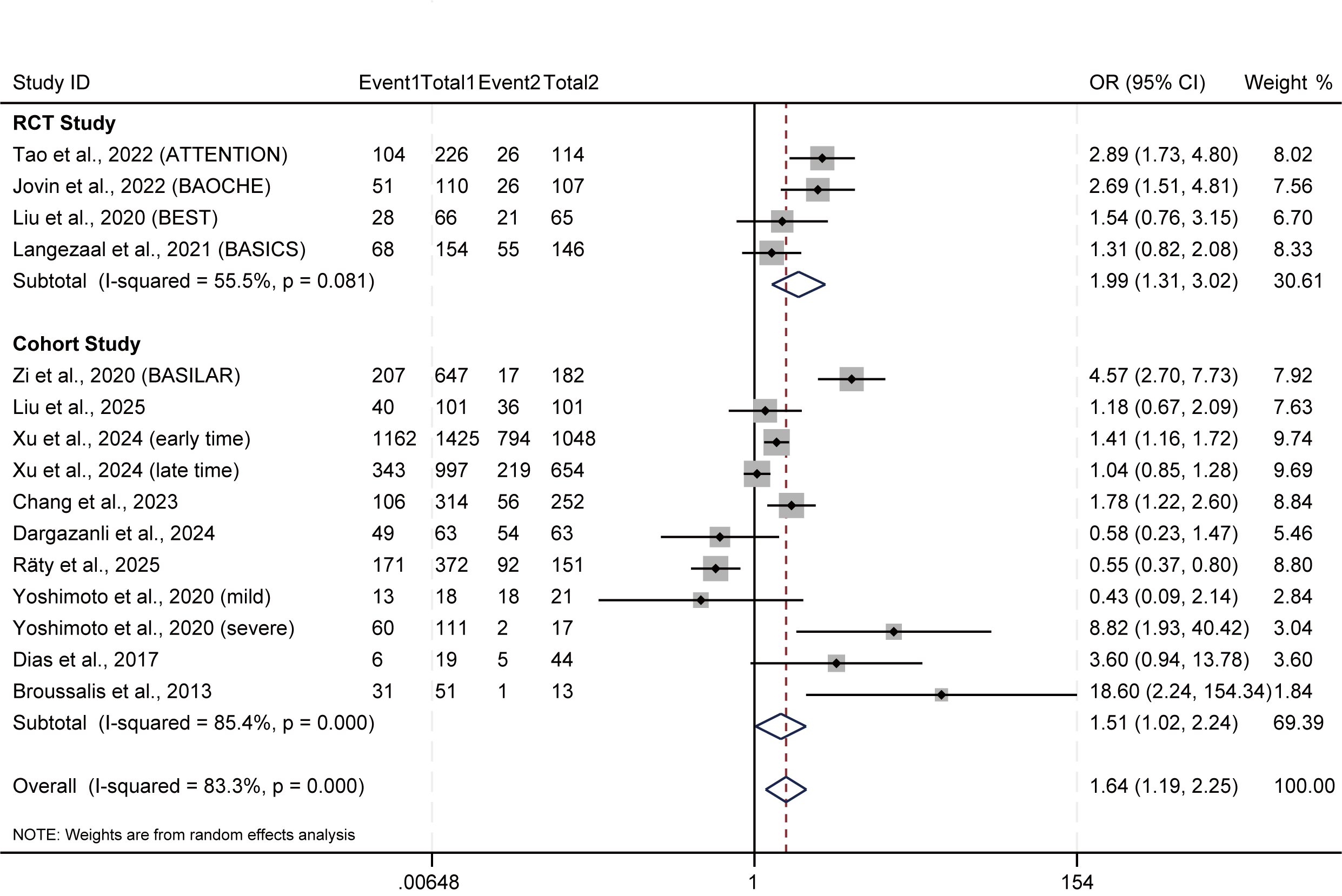
****

****
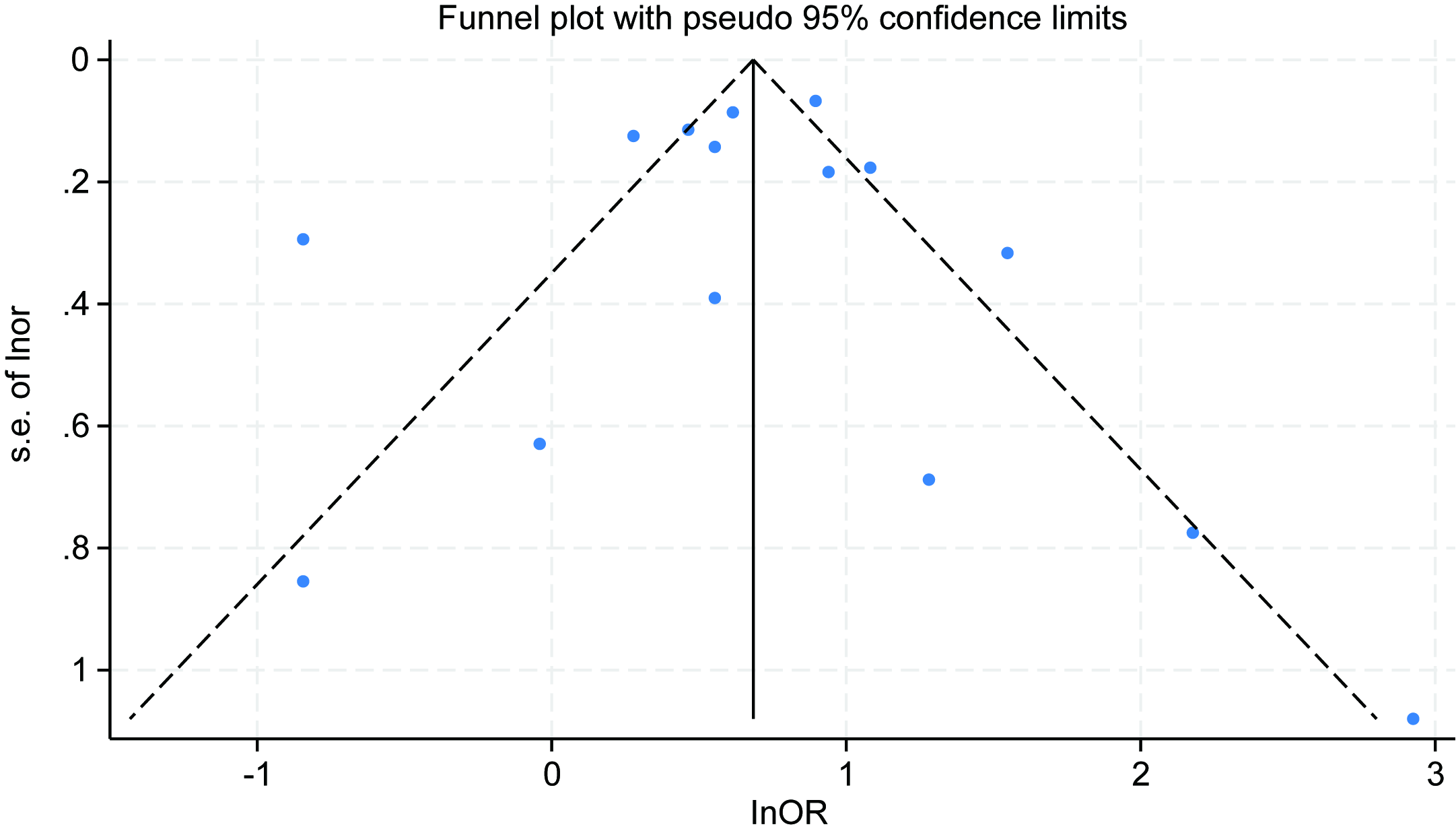
Supplementary Figure 6. Funnel Plot Assessing Publication Bias for the Primary Outcome (90-day mRS 0–3)****

****Supplementary Tables****

****Supplementary Table 1: Clinical Characteristics of Included Studies.****

| **Author (year)** | **Clinical history in EVT arm**  **no. (%)** | | | | | | |  | **Cause of stroke in EVT arm   no. (%)** | | |  | **Median Onset to revascularization EVT (min)** | **TICI score  of 2b or 3 in EVT arm no./total**  **no. (%)** |
| --- | --- | --- | --- | --- | --- | --- | --- | --- | --- | --- | --- | --- | --- | --- |
|  | **Hypertension** | **Atrial fibrillation** | **Smoking** | **Dyslipidemia** | **Diabetes** | **Stroke** | **Coronary Heart Disease** |  | **CE** | **LAA** | **Other or undetermined** |  |  |  |
| Tao et al., 2022 (ATTENTION) | 162 (72) | 45 (20) | 62 (27) | 61 (27) | 48 (21) | 50 （22.1） | 90 (40) |  | 46(20.0) | 108 (48) | 72(33.8) |  | 414 (360–528) | 208/223 (93.3) |
| Jovin et al., 2022 (BAOCHE) | 90 (82) | 14(13) | 34 (32) | 4 (4) | 30 (27) | 25 (22.7) | 15(13.6) |  | 11(10.0) | 75 (68.2) | 24(21.8) |  | 664 (512–861) | 63/88 (71.6) |
| Liu et al., 2020 (BEST) | 45 (68) | 18(27) | (33) 22 | 3 (5) | 10 (15) | 14(21) | 10 (15) |  | 10(15.0) | 37 (56.0) | 15(23.0) |  | 400 (269–526) | 45/63 (71.4) |
| Langezaal et al., 2021 (BASICS) | 93 (60) | 44 (29) | 64 (61) | 43 (32) | 32 (22) | 26(16.9) | 21 (13.6) |  | 51 (34.9) | 53(36.3) | 42(27.3) |  | NA | 63/77 (81.8) |
| Zi et al., 2020 (BASILAR) | 451(69.7) | 136 (21) | 235 (36.3) | 214(33.1) | 149 (23) | 140 (21.6) | 105(16.2) |  | 173（26.7） | 418（64.6） | 56（8.6） |  | 105 (71-151) | 522/647 (80.7) |
| Nicolin et al., 2024 | 261 (67.6) | 100 (25.9) | 82 (21.2) | 99 (25.6) | 76 (19.7) | 27 (7.0) | NA |  | NA | NA | NA |  | NA | 331/397 (83.4) |
| Liu et al., 2025 | 76 (75.3) | 14 (13.9) | 34(33.8) | 43 (42.6) | 36 (35.6) | 27(26.7) | 20(19.8) |  | 14 (13.9) | 84(83.7) | 3(3) |  | NA | NA |
| Xu et al., 2024 (early time) | 1040(73.0) | NA | NA | 501 (35.2) | 325 (22.8) | NA | NA |  | 337(23.6) | 902(63.3) | 186(13.1) |  | 105 (71-146) | NA |
| Xu et al., 2024 (late time) | 464 (46.5) | NA | NA | 359(36) | 226 (22.7) | NA | NA |  | 174(17.5) | 646(64.8) | 145(14.5) |  | 115 (80-167) | NA |
| Chang et al., 2023 | 217 (69.1) | 122 (38.9) | 113(36) | 88 (28) | 85(27.1) | 55 (17.5) | NA |  | 124 (39.5) | 102 (32.5) | 88 (28.0) |  | NA | NA |
| Dargazanli et al., 2024 | 47(74.6) | 23 (36.5) | NA | 23 (36.5) | 10(15.9) | NA | NA |  | NA | NA | NA |  | NA | NA |
| Yoshimoto et al., 2020 (mild) | 12 (67) | 9 (50) | 3 (17) | 5(28) | 4 (22) | 1(6) | NA |  | 13(72.2) | 4 (22.2) | 1 (6.0) |  | 55 (40- 105) | NA |
| Yoshimoto et al., 2020 (severe) | NA | 68 (61) | 60 (54) | 21 (19) | 32 (29) | 25(22) | 9(8) |  | 74 (66.7) | 27 (24.3) | 10(9.0) |  | 55（35 -80) | NA |
| Räty et al., 2025 | 263(70.7) | 94 (25.3) | NA | 138 (37.2) | 88(23.7) | 78(21.0) | 102 (27.4) |  | 126 (35.9) | 121 (34.5) | 108(30.8) |  | NA | NA |
| Seners et al., 2021 | 16 (55) | 4 (14) | 7(15) | NA | 2 (7) | NA | NA |  | NA | NA | NA |  | 298 (251-372) | 17/22(77) |
| Dias et al., 2017 | 14(74) | 2(11) | 7(37) | 6(32) | 2(11) | 4(21) | 1(5) |  | 6 (32) | 9 (47) | 4 (21) |  | 452 (302-625) | NA |
| Broussalis et al., 2013 | NA | NA | NA | NA | NA | NA | NA |  | NA | NA | NA |  | NA | 51/77(66.2) |

****Supplementary Table 2. Adjusted Variables for Confounding Control in the Included Studies****

| **Study name** | **Confounders adjustment** |
| --- | --- |
| Tao et al., 2022 (ATTENTION) | age, modified Rankin scale score before the stroke, time from stroke onset to randomization, and baseline stroke severity (NIHSS score). |
| Jovin et al., 2022 (BAOCHE) | age, baseline NIHSS score, and the time from stroke onset |
| Liu et al., 2020 (BEST) | age and baseline NIHSS score |
| Langezaal et al., 2021 (BASICS) | age, NIHSS and atrial fibrillation |
| Zi et al., 2020 (BASILAR) | age, baseline NIHSS score, baseline pc-ASPECTS, onset-to-imaging diagnosis time, sex, intravenous thrombolysis, diabetes mellitus, ischemic stroke, onset-to–outcome measurement time, and location of occlusion |
| Nicolin et al., 2024 | age, sex, onset-to-treatment time, hypertension, diabetes, hypercholesterolemia, history of TIA or stroke in the past 3 months, atrial fibrillation, heart failure, smoking habit, baseline NIHSS score, and site of BAO. |
| Liu et al., 2025 | sex, age, admission blood pressure, hypertension, atrial fibrillation, smoking, hypertriglyceridemia, diabetes, baseline NIHSS score, estimated time from basilar artery occlusion to admission, occlusion sites (VBAO with vs without vertebral artery occlusion), baseline CT pc-ASPECTS, baseline CT PMI, baseline MRI pc-ASPECTS, and baseline MRI PMI. |
| Xu et al., 2024 (early time) | age, sex, cerebrovascular risk factors, baseline NIHSS, pc-ASPECTS,stroke classification, collateral status, reperfusion status, IVT, and site of occlusion |
| Xu et al., 2024 (late time) | age, sex, cerebrovascular risk factors, baseline NIHSS, pc-ASPECTS,stroke classification, collateral status, reperfusion status, IVT, and site of occlusion |
| Chang et al., 2023 | age, sex, stroke severity, stroke subtype,prestroke mRS, stroke risk factors (e.g., prior history of stroke, hypertension, diabetes mellitus [DM], dys lipidemia, atrial fibrillation, smoking), PC-ASPECTS, |
| Dargazanli et al., 2024 | age,admission NIHSS |
| Räty et al., 2025 | age, baseline pc-ASPECTS, admission NIHSS |
| Seners et al., 2021 | age and pc-ASPECTS |

**Supplementary Table3: Basic Characteristics and Outcome Data of Included Studies**

| **Study ID** | **Study type** | **Outcome** | **OR(95% CI)** | **Source of OR** | **EVT No./Total No** | **SMT No./Total No** |
| --- | --- | --- | --- | --- | --- | --- |
| Tao et al., 2022 (ATTENTION) | RCT | mRS0-3 | 2.95(2.08,4.16) | Converted from aRR 2.06 (1.46,2.91) | 104/226 | 26/114 |
| Jovin et al., 2022 (BAOCHE) | RCT | mRS0-3 | 2.56(1.79,3.68) | Converted from aRR 1.81 (1.26,2.60) | 51/110 | 26/107 |
| Liu et al., 2020 (BEST) | RCT | mRS0-3 | 1.74 (0.81,3.74) | Directly extracted from original article | 28/66 | 21/65 |
| Langezaal et al., 2021 (BASICS) | RCT | mRS0-3 | 1.32(1.03,1.68) | Converted from aRR1.18(0.92,1.50) | 68/154 | 55/146 |
| Zi et al., 2020 (BASILAR) | PCS | mRS0-3 | 4.70 (2.53,8.75) | Directly extracted from original article | 207/647 | 17/182 |
| Liu et al., 2025 | PCS | mRS0-3 | 1.74(1.32,2.31) | Converted from PSM-adjusted RR1.35 (1.02 to 1.79) | 40/101(PSM 41/71) | 36/101(PSM 32/71) |
| Xu et al., 2024 (early time) | RCS | mRS0-3 | 2.45 (2.15,2.80) | Directly extracted PSM-adjusted OR from original article | 1162/1425(PSM NA/789) | 794/1048(PSM NA/789) |
| Xu et al., 2024 (late time) | RCS | mRS0-3 | 1.85 (1.57,2.20) | Directly extracted PSM-adjusted OR from original article | 343/997(PSM NA/482) | 219/654(PSM NA/482) |
| Chang et al., 2023 | RCS | mRS0-3 | 1.59(1.27,1.99) | Converted from IPTW-adjusted RR1.39 (1.11–1.74) | 106/314(IPTW 185/557) | 56/252 (IPTW 133/557) |
| Dargazanli et al., 2024 | RCS | mRS0-3 | 0.96（0.28, 3.30） | Directly extracted IPTW-adjusted OR from original article | 49/63(IPTW NA) | 54/63(IPTW NA) |
| Räty et al., 2025 | RCS | mRS0-3 | 0.43 (0.24,0.76) | Reciprocal of IPWRA OR2.33 (1.31-4.12) from original article | 171/372(IPWAR NA) | 92/151(IPWAR NA) |
| Yoshimoto et al., 2020 (mild) | RCS | mRS0-3 | 0.43 (0.08,2.28) | Unadjusted OR calculated from raw counts | 13/18 | 18/21 |
| Yoshimoto et al., 2020 (severe) | RCS | mRS0-3 | 8.82 (1.93,40.25) | Unadjusted OR calculated from raw counts | 60/111 | 2/ 17 |
| Dias et al., 2017 | RCS | mRS0-3 | 3.6(0.93,13.79) | Unadjusted OR calculated from raw counts | 6/19 | 5/44 |
| Broussalis et al., 2013 | RCS | mRS0-3 | 18.64(2.24,154.34) | Unadjusted OR calculated from raw counts | 31/51 | 1/13 |
| Tao et al., 2022 (ATTENTION) | RCT | mRS0-2 | 4.22(2.45,7.33) | Converted from aRR 3.17 (1.84 to 5.46) | 75/226 | 12/114 |
| Jovin et al., 2022 (BAOCHE) | RCT | mRS0-2 | 3.90(2.34,6.46) | Converted from aRR 2.75 (1.65 to 4.56) | 43/110 | 15/107 |
| Liu et al., 2020 (BEST) | RCT | mRS0-2 | 1.40(0.64,3.10) | Directly extracted from original article | 22/66 | 18/65 |
| Langezaal et al., 2021 (BASICS) | RCT | mRS0-2 | 1.26(0.94,1.70) | Converted from aRR1.17 (0.87 to 1.57) | 54/154 | 44/146 |
| Zi et al., 2020 (BASILAR) | PCS | mRS0-2 | 4.90 (2.43,9.87) | Directly extracted multivariable-adjusted OR from primary analysis in original article. | 177/647 | 13/182 |
| Liu et al., 2025 | PCS | mRS0-2 | 1.26(0.88,1.81) | Converted from PSM-adjusted RR 1.29 (0.90 to 1.85) | 19/101(PSM 28/71) | 27/101(PSM 29/71) |
| Xu et al., 2024 (early time) | RCS | mRS0-2 | 1.95(1.72,2.21) | Directly extracted PSM-adjusted OR from original article | 858/1425(PSM NA/789) | 626/1048(PSM NA/789) |
| Xu et al., 2024 (late time) | RCS | mRS0-2 | 1.49(1.26,1.76) | Directly extracted PSM-adjusted OR from original article | 305/997(PSM NA/482) | 177/654(PSM NA/482) |
| Chang et al., 2023 | RCS | mRS0-2 | 1.38(1.06,1.82) | Converted from IPTW-adjusted RR1.30 (0.99–1.71) | 70/314(IPTW 120/557) | 38/252(IPTW 92/557) |
| Dargazanli et al., 2024 | RCS | mRS0-2 | 1.49(0.55,4.05) | Directly extracted IPTW-adjusted OR from original article | 43/63(IPTW NA) | 45/63(IPTW NA) |
| Räty et al., 2025 | RCS | mRS0-2 | 0.52 (0.30–0.89) | Reciprocal of IPWRA OR1.93 (1.12–3.30) | 128/372(IPWAR NA) | 70/151(IPWAR NA) |
| Nicolin et al., 2024 | PCS | mRS0-2 | 1.56 (1.04–2.03) | Directly extracted IPW-adjusted OR from original article | 261/410(IPW 454/710) | 213/345(IPW 383/707) |
| Seners et al., 2021 | RCS | mRS0-2 | 5.11(1.27,20.58) | Directly extracted PSM-adjusted OR from original article | 25/28(PSM NA) | 21/29(PSM NA） |
| Yoshimoto et al., 2020 (mild) | RCS | mRS0-2 | 0.37 (0.09–1.57) | Unadjusted OR calculated from raw counts | 11/18 | 17/21 |
| Yoshimoto et al., 2020 (severe) | RCS | mRS0-2 | 5.52 (1.23–24.80) | Unadjusted OR calculated from raw counts | 47/111 | 2/77 |
| Broussalis et al., 2013 | RCS | mRS0-2 | 22.26(1.26,394.63) | Unadjusted OR calculated from raw counts | 23/51 | 0/13 |
| Tao et al., 2022 (ATTENTION) | RCT | Mortality | 0.46(0.36,0.58) | Converted from aRR 0.66 (0.52 to 0.82) | 83/226 | 63/114 |
| Jovin et al., 2022 (BAOCHE) | RCT | Mortality | 0.62(0.45,0.87) | Converted from aRR 0.75 (0.54 to 1.04) | 34/110 | 45/107 |
| Liu et al., 2020 (BEST) | RCT | Mortality | 0.80 (0.37,1.64) | Directly extracted from original article | 22/66 | 25/65 |
| Langezaal et al., 2021 (BASICS) | RCT | Mortality | 0.80(0.63,1.03) | Converted from aRR0.87 (0.68 to 1.12) | 59/154 | 63/146 |
| Zi et al., 2020 (BASILAR) | PCS | Mortality | 0.34(0.23,0.51) | Reciprocal of 2.93 (1.95-4.40) from original article | 299/647 | 130/182 |
| Liu et al., 2025 | PCS | Mortality | 0.22(0.07,0.66) | Converted from PSM-adjusted 0.27 (0.08 to 0.81) | 18/101(PSM 9/71) | 26/101(PSM 20/71) |
| Xu et al., 2024 (early time) | RCS | Mortality | 0.54(0.48-0.61) | Directly extracted PSM-adjusted OR from original article | 500/1425(PSM NA/789) | 447/1048(PSM NA/789) |
| Xu et al., 2024 (late time) | RCS | Mortality | 0.59(0.50-0.69) | Directly extracted PSM-adjusted OR from original article | 388/997(PSM NA/482) | 301/654(PSM NA/482) |
| Chang et al., 2023 | RCS | Mortality | 0.44(0.36,0.54) | Converted from IPTW-adjusted RR0.60 (0.49–0.73) | 85/314(IPTW 157/557) | 114/252(IPTW 263/557) |
| Dargazanli et al., 2024 | RCS | Mortality | 1.49(0.37,5.95) | Directly extracted IPTW-adjusted OR from original article | 9/63(IPTW NA) | 4/63(IPTW NA) |
| Räty et al., 2025 | RCS | Mortality | 1.89 (1.03–3.45) | Reciprocal of IPWRA OR0.53 (0.29–0.97) | 150/372(IPWAR NA) | 45/151(IPWAR NA) |
| Nicolin et al., 2024 | PCS | Mortality | 0.65 (0.42–1.01) | Directly multivariable-adjusted OR from original article | 73/410(IPW 112/710) | 69/345(IPW 156/710) |
| Yoshimoto et al., 2020 (mild) | RCS | Mortality | 0.39 (0.02,9.87) | Unadjusted OR calculated from raw counts | 0/18 | 1 21 |
| Yoshimoto et al., 2020 (severe) | RCS | Mortality | 0.29 (0.10,0.86) | Unadjusted OR calculated from raw counts | 15/111 | 6/17 |
| Dias et al., 2017 | RCS | Mortality | 0.31(0.10,0.93) | Unadjusted OR calculated from raw counts | 8/19 | 31/44 |
| Broussalis et al., 2013 | RCS | Mortality | 0.98(0.41,2.37) | Unadjusted OR calculated from raw counts | 31/77 | 9/22 |

**Supplementary Table 4: Subgroup-Specific Relative Risk (RR) of Outcomes Across Studies**

| **Subgroup** | **Study** | **RR** | **lci** | **Uci** | **Data Type** |
| --- | --- | --- | --- | --- | --- |
| age＜75 | Zi et al., 2020 (BASILAR) | 4.28 | 1.71 | 10.64 | PSM-matched (RR) |
| age＜75 | Liu et al., 2025 | 1.17 | 0.64 | 2.11 | aOR≈aRR |
| age＜75 | Tao et al., 2022 (ATTENTION) | 2.01 | 1.32 | 3.04 | aRR |
| age＜75 | Langezaal et al., 2021 (BASICS) | 1.03 | 0.77 | 1.37 | aRR |
| age＜75 | Jovin et al., 2022 (BAOCHE) | 1.7 | 1.17 | 2.48 | aRR |
| age≥75 | Chang et al., 2023 | 5.14 | 2.29 | 11.55 | aRR |
| age≥75 | Tao et al., 2022 (ATTENTION) | 2.45 | 0.6 | 9.99 | aRR |
| age≥75 | Xu et al., 2024 | 0.68 | 0.46 | 1.02 | aOR≈RR |
| age≥75 | Liu et al., 2025 | 2.38 | 0.44 | 15 | aOR≈RR |
| Female | Zi et al., 2020 (BASILAR) | 2.33 | 1.19 | 4.57 | PSM-matched (RR) |
| Female | Xu et al., 2024 | 0.82 | 0.58 | 1.14 | aOR≈RR |
| Female | Chang et al., 2023 | 1.65 | 1.1 | 2.46 | aRR |
| Female | Tao et al., 2022 (ATTENTION) | 1.7 | 0.98 | 2.94 | aRR |
| Female | Jovin et al., 2022 (BAOCHE) | 2.95 | 1.14 | 7.59 | aRR |
| Male | Zi et al., 2020 (BASILAR) | 3.52 | 1.51 | 8.21 | PSM-matched (RR) |
| Male | Xu et al., 2024 | 0.9 | 0.73 | 1.09 | aOR≈RR |
| Male | Chang et al., 2023 | 1.28 | 0.98 | 1.68 | aRR |
| Male | Tao et al., 2022 (ATTENTION) | 2.13 | 1.39 | 3.26 | aRR |
| Male | Jovin et al., 2022 (BAOCHE) | 1.61 | 1.09 | 2.36 | aRR |
| NIHSS＜10 | Liu et al., 2025 | 1.12 | 0.33 | 3.99 | aOR≈RR |
| NIHSS＜10 | Chang et al., 2023 | 1.15 | 0.85 | 1.57 | aRR |
| NIHSS＜10 | Räty et al., 2025 | 0.47 | 0.31 | 0.72 | aOR≈aRR |
| NIHSS＜10 | Langezaal et al., 2021 (BASICS) | 0.85 | 0.62 | 1.16 | aRR |
| NIHSS＜10 | Yoshimoto et al., 2020 | 0.84 | 0.58 | 1.22 | raw data-derived RR |
| NIHSS≥10 | Liu et al., 2025 | 5.93 | 2.47 | 15.5 | aOR≈RR |
| NIHSS≥10 | Chang et al., 2023 | 2.01 | 1.42 | 2.83 | aRR |
| NIHSS≥10 | Räty et al., 2025 | 0.64 | 0.43 | 0.95 | aOR≈RR |
| NIHSS≥10 | Yoshimoto et al., 2020 | 4.59 | 1.23 | 17.15 | raw data-derived RR |
| NIHSS≥10 | Zi et al., 2020 (BASILAR) | 3.19 | 1.08 | 9.45 | PSM-matched (RR) |
| NIHSS≥10 | Xu et al., 2024 | 0.9 | 0.73 | 1.09 | aOR≈RR |
| NIHSS≥10 | Tao et al., 2022 (ATTENTION) | 2.06 | 1.46 | 2.91 | aRR |
| NIHSS≥10 | Langezaal et al., 2021 (BASICS) | 1.45 | 1.03 | 2.04 | aRR |
| NIHSS≥10 | Jovin et al., 2022 (BAOCHE) | 2 | 1.23 | 3.25 | aRR |
| NIHSS≥10 | Jovin et al., 2022 (BAOCHE) | 1.83 | 0.73 | 4.58 | aRR |
| pc-ASPECTS＜8 | Zi et al., 2020 (BASILAR) | 2.61 | 1.5 | 4.54 | PSM-matched (RR) |
| pc-ASPECTS＜8 | Chang et al., 2023 | 3.07 | 1.55 | 6.09 | aRR |
| pc-ASPECTS＜8 | Tao et al., 2022 (ATTENTION) | 3.86 | 0.98 | 15.24 | aRR |
| pc-ASPECTS＜8 | Langezaal et al., 2021 (BASICS) | 1.08 | 0.41 | 2.83 | aRR |
| pc-ASPECTS＜8 | Liu et al., 2025 | 1.54 | 0.59 | 4.03 | aRR |
| pc-ASPECTS≥8 | Liu et al., 2025 | 1.96 | 0.20 | 6.28 | aOR≈aRR |
| pc-ASPECTS≥8 | Xu et al., 2024 | 0.92 | 0.65 | 1.30 | aOR≈aRR |
| pc-ASPECTS≥8 | Jovin et al., 2022 (BAOCHE)1 | 1.42 | 0.86 | 2.34 | aRR |
| pc-ASPECTS≥8 | Jovin et al., 2022 (BAOCHE)2 | 2.17 | 1.28 | 3.66 | aRR |
| pc-ASPECTS≥8 | Zi et al., 2020 (BASILAR) | 2.72 | 0.99 | 7.45 | PSM-matched (RR) |
| pc-ASPECTS≥8 | Tao et al., 2022 (ATTENTION) | 1.94 | 1.37 | 2.74 | aRR |
| pc-ASPECTS≥8 | Langezaal et al., 2021 (BASICS) | 1.16 | 0.89 | 1.51 | aRR |
| IVT-YES | Zi et al., 2020 (BASILAR) | 2.15 | 0.85 | 5.4 | PSM-matched (RR) |
| IVT-YES | Xu et al., 2024 | 0.85 | 0.56 | 1.28 | aOR≈aRR |
| IVT-YES | Chang et al., 2023 | 0.89 | 0.64 | 1.24 | aRR |
| IVT-YES | Tao et al., 2022 (ATTENTION) | 1.57 | 0.97 | 2.54 | aRR |
| IVT-YES | Langezaal et al., 2021 (BASICS)1 | 1.16 | 0.88 | 1.53 | aRR |
| IVT-YES | Langezaal et al., 2021 (BASICS)2 | 2.7 | 0.9 | 8.11 | aRR |
| IVT-NO | Zi et al., 2020 (BASILAR) | 3.13 | 1.68 | 5.79 | PSM-matched (RR) |
| IVT-NO | Xu et al., 2024 | 0.92 | 0.76 | 1.11 | aOR≈aRR |
| IVT-NO | Chang et al., 2023 | 2 | 1.46 | 2.73 | aRR |
| IVT-NO | Tao et al., 2022 (ATTENTION) | 2.56 | 1.56 | 4.18 | aRR |
| IVT-NO | Langezaal et al., 2021 (BASICS) | 1.08 | 0.53 | 2.23 | aRR |
| BA distal | Zi et al., 2020 (BASILAR) | 2.86 | 1.39 | 6.62 | PSM-matched (RR) |
| BA distal | Tao et al., 2022 (ATTENTION) | 1.33 | 0.86 | 2.03 | aRR |
| BA distal | Langezaal et al., 2021 (BASICS) | 0.98 | 0.73 | 1.33 | aRR |
| BA middle | Zi et al., 2020 (BASILAR) | 2.83 | 1.39 | 6.62 | PSM-matched (RR) |
| BA middle | Tao et al., 2022 (ATTENTION) | 2.06 | 0.92 | 4.63 | aRR |
| BA middle | Langezaal et al., 2021 (BASICS) | 1.24 | 0.78 | 1.95 | aRR |
| BA middle | Jovin et al., 2022 (BAOCHE) | 1.67 | 0.87 | 3.22 | aRR |
| BA proximal | Zi et al., 2020 (BASILAR) | 2.5 | 0.63 | 8.26 | PSM-matched (RR) |
| BA proximal | Tao et al., 2022 (ATTENTION) | 3.09 | 1.45 | 6.58 | aRR |
| BA proximal | Langezaal et al., 2021 (BASICS) | 1.59 | 0.84 | 3.03 | aRR |
| BA proximal | Jovin et al., 2022 (BAOCHE) | 1.96 | 1.15 | 3.36 | aRR |
| VA | Zi et al., 2020 (BASILAR) | 2.5 | 0.73 | 7.17 | PSM-matched (RR) |
| VA | Liu et al., 2025 | 1.95 | 0.82 | 4.65 | aOR≈aRR |
| OTA≤6h | Zi et al., 2020 (BASILAR) | 2.76 | 1.73 | 5.42 | PSM-matched (RR) |
| OTA≤6h | Chang et al., 2023 | 1.08 | 0.82 | 1.43 | aRR |
| OTA≤6h | Räty et al., 2025 | 0.37 | 0.18 | 0.74 | aOR≈aRR |
| OTA≤6h | Tao et al., 2022 (ATTENTION) | 1.94 | 1.26 | 2.98 | aRR |
| OTA≤6h | Xu et al., 2024(1) | 2.16 | 1.94 | 2.41 | aOR≈aRR |
| OTA≤6h | Langezaal et al., 2021 (BASICS) | 1.18 | 0.92 | 1.50 | aRR |
| OTA>6h | Zi et al., 2020 (BASILAR) | 2.86 | 0.89 | 7.39 | PSM-matched (RR) |
| OTA>6h | Liu et al., 2025 | 1.35 | 1.02 | 1.79 | aRR |
| OTA>6h | Chang et al., 2023 | 2.11 | 1.45 | 3.07 | aRR |
| OTA>6h | Jovin et al., 2022 (BAOCHE) | 1.71 | 1.01 | 2.90 | aRR |
| OTA>6h | Xu et al., 2024(2) | 1.89 | 1.65 | 2.17 | aOR≈aRR |
| OTA>6h | Räty et al., 2025 | 0.77 | 0.24 | 2.44 | aOR≈aRR |
|  |  |  |  |  |  |

**Supplementary Table 5. Risk of Bias Assessment of RCTs (Cochrane RoB 2.0 Tool)**

| **Study ID, year** | **D1** | **D2** | **D3** | **D4** | **D5** | **Overall** |
| --- | --- | --- | --- | --- | --- | --- |
| Tao et al., 2022 (ATTENTION) | ＋ | ＋ | ＋ | ＋ | ＋ | ＋ |
| Jovin et al., 2022 (BAOCHE) | ＋ | ＋ | ＋ | ＋ | ＋ | ＋ |
| Liu et al., 2020 (BEST) | ＋ | X | ＋ | ＋ | ＋ | X |
| Langezaal et al., 2021 (BASICS) | ＋ | － | ＋ | ＋ | ＋ | － |

Domains: D1: Randomization process; D2: Deviations from intended interventions; D3: Missing outcome data; D4: Outcome measurement; D5: Selective reporting.
Risk of bias legend: + Low risk; - Some concerns; X High risk.

****Supplementary Table 6. Quality Assessment of Observational Cohort Studies (Newcastle-Ottawa Scale)****

| **Study ID, year** | **Selection** | **Comparability** | **Outcome** | **Overall score** |
| --- | --- | --- | --- | --- |
| Zi et al., 2020 (BASILAR) | 3* | 2* | 3* | 8 |
| Nicolin et al., 2024 | 3* | 2* | 3* | 8 |
| Liu et al., 2025 | 4* | 2* | 3* | 9 |
| Xu et al., 2024 (early time) | 3* | 2* | 3* | 8 |
| Xu et al., 2024 (late time) | 3* | 2* | 3* | 8 |
| Chang et al., 2023 | 3* | 2* | 3* | 8 |
| Dargazanli et al., 2024 | 3* | 2* | 3* | 8 |
| Yoshimoto et al., 2020 (mild) | 3* | 2* | 3* | 8 |
| Yoshimoto et al., 2020 (severe) | 3* | 2* | 3* | 8 |
| Räty et al., 2025 | 4* | 2* | 3* | 9 |
| Seners et al., 2021 | 3* | 2* | 3* | 8 |
| Dias et al., 2017 | 3* | 1* | 3* | 7 |
| Broussalis et al., 2013 | 3* | 1* | 3* | 7 |

A score of ≥7 stars is generally considered indicative of high methodological quality.
